# Editorial: Veterinary Sports Medicine and Physical Rehabilitation

**DOI:** 10.3389/fvets.2020.00240

**Published:** 2020-04-28

**Authors:** David Levine, Henry Steven Adair, Denis J. Marcellin-Little, Michael Jaffe, Andris J. Kaneps

**Affiliations:** ^1^Department of Physical Therapy, University of Tennessee at Chattanooga, Chattanooga, TN, United States; ^2^Department of Large Animal Clinical Sciences, The University of Tennessee, Knoxville, Knoxville, TN, United States; ^3^Department of Surgical and Radiological Sciences, University of California, Davis, Davis, CA, United States; ^4^Department of Clinical Sciences, College of Veterinary Medicine, Mississippi State University, Starkville, MS, United States; ^5^Kaneps Equine Sports Medicine and Surgery, Beverly, MA, United States

**Keywords:** rehabilitation, physical therapy, sports medicine, orthopedics, neurology

There is a clear need and a strong interest on the part of the veterinary profession to learn more about sports medicine and physical rehabilitation following injury, surgery, and illness, and to optimize patient outcomes by incorporating physical rehabilitation into practice. Human sports medicine and rehabilitation is a well-established discipline whose positive benefits have been clearly documented and recognized in human health care. Historically, relatively little attention was given to veterinary patients afflicted with similar conditions. The techniques used in human sports medicine and physical therapy are being adapted for use in small animal and equine patients, and their effectiveness has been or is being studied. The growing interest in sports medicine and physical rehabilitation among veterinarians has led to the formation of a specialty College, the American College of Veterinary Sports Medicine and Rehabilitation in 2010 and a veterinary technician specialty, the Academy of Physical Rehabilitation Veterinary Technicians in 2017.

This Research Topic issue seeks to address the science of small animal and equine sports medicine and rehabilitation to help provide better understanding of assessment methods, treatment techniques, and interventions utilized. Forty authors contributed to the 10 articles published in this issue.

A number of outcome measures are used to assess dogs during rehabilitation. Goniometry being one of the most widely used methods. Formenton et al. examined normal joint angles and range of motion in French Bulldogs using the standard method first described and published in 2002 (1) to examine how this breed might differ from others previously examined. Fahie et al. also studied an outcome measure to help us obtain better information on gait in dogs. The system they studied on 66 dogs discusses a simple and reliable gait assessment method that can be implemented in clinical practice.

Another assessment method was detailed in a paper entitled Variables Affecting Thigh Girth Measurement and Observer Reliability in Dogs by McCarthy et al. Measurement of muscle girth to indirectly assess muscle mass has been used for document muscle atrophy in patients and the recovery of muscle mass in response to rehabilitation. This study evaluated the use of thigh girth measurement in dogs before and after surgery of the stifle joint and evaluated inter-rater reliability which was excellent when conditions were standardized.

Prosthetics has been an evolving field in canine rehabilitation, and socket prostheses have been a more viable treatment option for distal limb pathologies including amputation (2). Wendland et al. performed a multi-center study evaluating owner satisfaction with socket prosthesis use in dogs. The authors found that owners were very satisfied, despite the presence of a substantial complication rate.

Laser therapy is a popular treatment modality in canine rehabilitation and sports medicine and one of the conditions it is used for is wound healing. The optimal parameters including dosage for wound healing is unknown but studies such as the one by Wardlaw et al. provide guidance for future work. In this study, they found that daily application of laser therapy at 8 J/cm^2^ hastened wound healing in Dachshunds that underwent thoracolumbar hemilaminectomies to manage intervertebral disc disease.

Therapeutic exercises are an essential part of rehabilitation and sports medicine and surface electromyography (sEMG) provides a way of objectively measuring muscle activity during exercise. A prospective trial by McLean et al. continues to build on the previous sEMG work in dogs (3, 4) and adds to our knowledge base. The objective of the study reported here was to analyze the mean and maximum muscle activation patterns of the vastus lateralis, biceps femoris, and gluteus medius during stance, walking, trotting, and specific therapeutic exercises in clinically sound, healthy dogs. These results may help clinicians to choose specific exercises to target specific muscles during conditioning, strengthening, and rehabilitation.

Two of the studies in this Research Topic issue focused on therapeutic ultrasound which has been used in rehabilitation and sports medicine for humans, dogs, and horses (5). The study on dogs investigated by Acevedo et al.. This prospective, crossover, experimental study concluded that the heating effects of therapeutic ultrasound increase the effectiveness of stretching connective tissues, but these effects are short lived. This is in agreement with the human literature. The equine study by Adair and Levine presented here continues work performed by Levine et al. (6) and Montgomery et al. (7) The main findings of the study is that use of therapeutic ultrasound with a 1.0 MHz US for 10 min in horse's epaxial muscles when clipped creates the greatest heat at 1.0 cm depth. The heat in tissues at a 5-cm depth is more than at a 3-cm depth.

Wilson et al., in the article titled International Survey Regarding the Use of Rehabilitation Modalities in Horses attempted to define which biologic, electrophysical, and other modalities are used in horses for injury or performance issues. To achieve this, the authors developed a questionnaire listing 38 modalities and distributed it to eight veterinary groups. Their findings indicate that a broad range of invasive and non-invasive modalities are used in equine patients to address a variety of rehabilitation and performance needs, and that personnel with varying levels of expertise are involved in their administration.

Riccio et al., in the article titled Two Multicenter Surveys on Equine Back-Pain 10 Years Apart endeavored to assess the evolution in the veterinarian approach to diagnose and treat back-pain over a 10 years period. To investigate this topic, two surveys were sent to equine veterinarians working in practice throughout Europe 10 years apart, in 2006 and 2016. The study provided insight into the current perception of clinicians working in different settings regarding horse back-pain but did not identify changes in veterinarians' approaches to the diagnosis and management of equine back pain over the last decade.

## Author Contributions

All authors listed have made a substantial, direct and intellectual contribution to the work, and approved it for publication.

## Conflict of Interest

The authors declare that the research was conducted in the absence of any commercial or financial relationships that could be construed as a potential conflict of interest.
